# Multiparametric MRI Features Predict the SYP Gene Expression in Low-Grade Glioma Patients: A Machine Learning-Based Radiomics Analysis

**DOI:** 10.3389/fonc.2021.663451

**Published:** 2021-05-31

**Authors:** Zheng Xiao, Shun Yao, Zong-ming Wang, Di-min Zhu, Ya-nan Bie, Shi-zhong Zhang, Wen-li Chen

**Affiliations:** ^1^Department of Neurosurgery, Zhujiang Hospital, Southern Medical University, Guangzhou, China; ^2^Center for Pituitary Tumor Surgery, Department of Neurosurgery, The First Affiliated Hospital of Sun Yat-sen University, Guangzhou, China; ^3^Center for Skull Base Surgery, Department of Neurosurgery, Brigham and Women’s Hospital, Harvard Medical School, Boston, MA, United States; ^4^School of Life Sciences and Biopharmaceutics, Guangdong Pharmaceutical University, Guangzhou, China

**Keywords:** synaptophysin (SYP), MRI radiomics model, convolutional neural network, glioma, machine learning

## Abstract

**Purpose:**

Synaptophysin (SYP) gene expression levels correlate with the survival rate of glioma patients. This study aimed to explore the feasibility of applying a multiparametric magnetic resonance imaging (MRI) radiomics model composed of a convolutional neural network to predict the SYP gene expression in patients with glioma.

**Method:**

Using the TCGA database, we examined 614 patients diagnosed with glioma. First, the relationship between the SYP gene expression level and outcome of survival rate was investigated using partial correlation analysis. Then, 7266 patches were extracted from each of the 108 low-grade glioma patients who had available multiparametric MRI scans, which included preoperative T1-weighted images (T1WI), T2-weighted images (T2WI), and contrast-enhanced T1WI images in the TCIA database. Finally, a radiomics features-based model was built using a convolutional neural network (ConvNet), which can perform autonomous learning classification using a ROC curve, accuracy, recall rate, sensitivity, and specificity as evaluation indicators.

**Results:**

The expression level of SYP decreased with the increase in the tumor grade. With regard to grade II, grade III, and general patients, those with higher SYP expression levels had better survival rates. However, the SYP expression level did not show any significant association with the outcome in Level IV patients.

**Conclusion:**

Our multiparametric MRI radiomics model constructed using ConvNet showed good performance in predicting the SYP gene expression level and prognosis in low-grade glioma patients.

## Introduction

In 2016, the World Health Organization (WHO) updated the tumor classification in the central nervous system and precisely introduced several molecular biomarkers that were integrated into the diagnostic criteria of glioma along with conventional histopathological diagnosis, aiding the advancement of precise diagnosis in glioma (1, 2). Likewise, under the guidance of molecular typing, the precise treatment of glioma has also been considerably expanded (3). Given these significant molecular markers, detecting them early and quickly has become extremely crucial.

Synaptophysin, the most commonly expressed neural marker, exists widely in a variety of lesions of primary central nervous system neoplasms, from gliomas to the lowest differentiated primitive neuroectodermal tumors (4, 5). The higher the degree of dedifferentiation of the tumor, the higher is the malignant degree. Therefore, as the most common neural marker, it is worth exploring whether the expressive level of synaptophysin is related to the malignant degree of gliomas and the survival prognosis of patients (6, 7).

In recent years, with the dramatic expansion of medical image analysis technology, radiomics has become a promising technique to bridge the gap between universal images and histopathological or molecular signatures (8). From medical images, a large number of high-throughput imaging features, including the extraction of tumor characteristics, can be used to quickly obtain heterogeneous information about tumors in a non-invasive manner (9, 10). The radiomics model established using machine learning has a high predictive potential and has been widely used for the precise prediction of various molecular types of glioma (11–13).

In this study, we used a convolutional neural network (ConvNet) to build a radiomics model based on multiparametric magnetic resonance imaging (MRI) to predict SYP expression levels in patients with low-grade glioma. The model is aimed at facilitating the implementation of molecular diagnosis in the early preoperative stage and the individualized treatment for patients with glioma.

## Materials and Methods

### Data Acquisition and Annotation

The imaging data and corresponding TCGA sequencing data of 124 patients with low-grade gliomas (WHOII, WHOIII) were downloaded from the TCIA. As the patients’ private information was de-identified by the TCGA/TCIA organization and their information was made available for download by the public, we did not have to apply for the approval of the Institutional Review Board or the health organizations following the Health Insurance Portability and Accountability Act.

The image were acquired using a 3.0-T MRI (Achieva, Philips). The T1WI(TR, 2000 ms; TE, 10 ms; FOV, 240 mm; slice thickness, 5 mm; and matrix size, 256 × 256), T2WI(TR, 3000 ms; TE, 80 ms; FOV, 240 mm; slice thickness, 5 mm; and matrix size, 256 × 256), and T1WI-enhanced (TR, 6.3 ms; TE, 3.1 ms) cross-sectional images of the tumor were imported into the 3D slicer analysis software in the Nifti format (14). Two neurosurgeons with over 10 years of working experience manually outlined the region of interest (ROI) along the tumor contour under double-blind conditions. The ROI included tumor parenchyma, necrosis, and cystic area, as well as surrounding edema. After finishing the outlining, the neurosurgeons analyzed the accuracy of the ROI and adjusted it after negotiating for the parts in dispute.

Images were re-sampled by the PyRadiomics toolkit (Version2.1.0, https://github.com/Radiomics/pyradiomics) to guarantee a 1.0 mm pixels interval among images on 3 anatomical directions, eliminating inconsistent spatial resolutions’ interference caused by the use of different models of MRI machine. Meanwhile, z-score normalization was applied to normalize the T1, T2, and T1E images, thereby obtaining the standard normal distribution of image intensity.

The transcriptome expression data of 614 gliomas were collected and downloaded online (http://cancergenome.nih.gov), ranging from WHO grade II to grade IV (150 GBM and 464 LGG samples). Information on age, sex, diagnosis, WHO grade, molecular data, and the patient prognosis was also collected. Patients were selected and grouped according to their median SYP expression (15, 16).

### Model Establishment and Performance Evaluation

Considering the shortcomings of traditional machine learning techniques, such as insufficient performance in classifying brain tumors, high complexity of manual feature extraction, and network degradation of conventional deep learning in deep-going networks, an automatic model of classifying brain tumors based on the ResNet50 network is proposed in this paper. First, the weight parameters of the model are obtained by training the source data, and then the performance of the model is tested using the test set.

**Figure 1:**
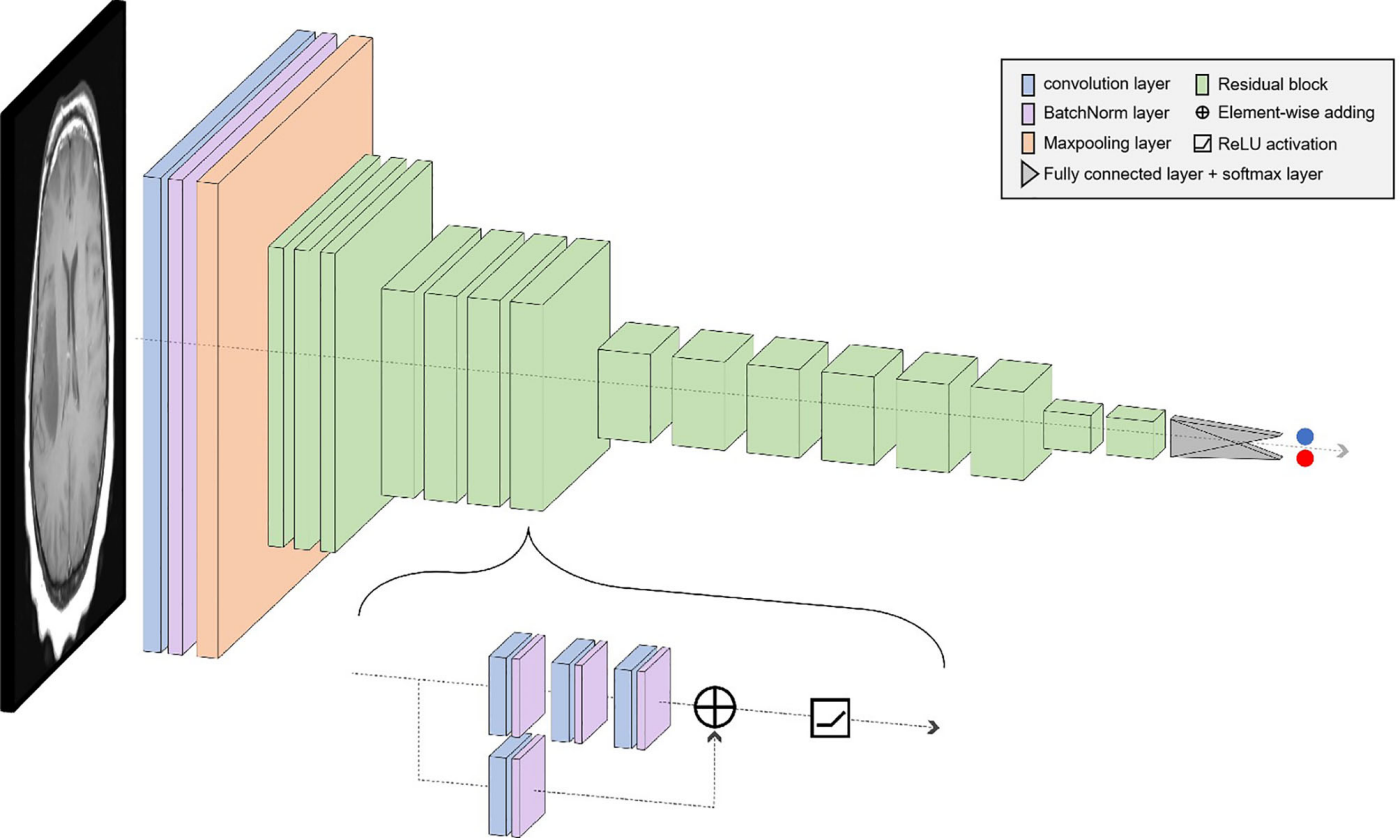
The learning framework of the ResNet50.

**Figure 2:**
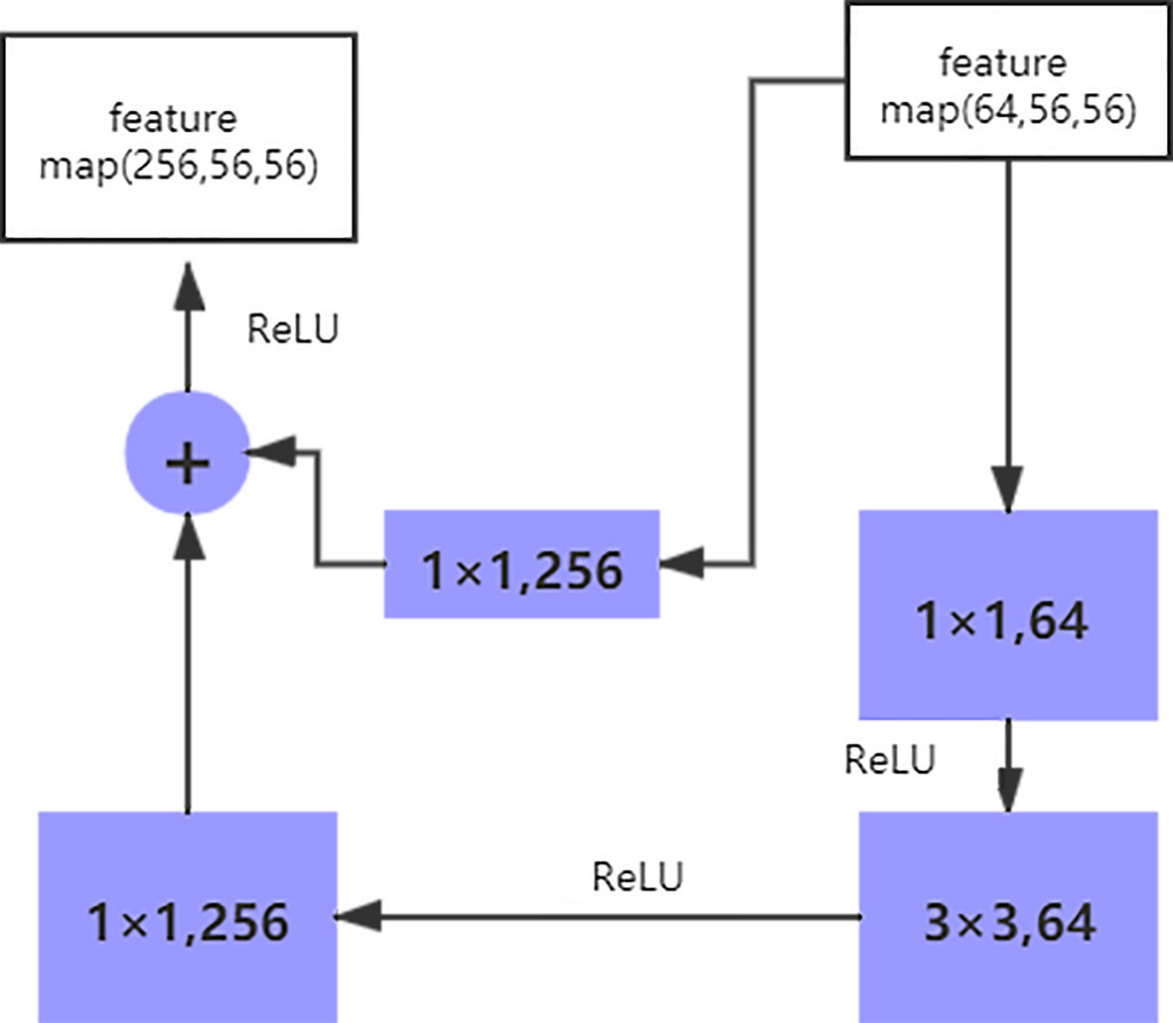
The structure of the residual block of the ResNet.

In deep learning, the main problems associated with network depth are gradient vanishing and gradient exploding. The traditional solution is to initialize and regularize the data, which deepens the depth and addresses the problem of the gradient but leads to the degradation of network performance. ResNet50 is a residual learning framework based on the existing deep network of training, which is easy to optimize and has the advantage of a small computational burden. Residuals are designed to address the problems of degradation and gradient, as a result of which the performance of the network improves. There are 49 convolutional layers and 1 fully connected layer in ResNet 50. Among them, the ID Block x2 in the second to the fifth stages represents two residual blocks that do not change the dimension, and the Conv Block represents the residual block with the dimension. Each residual Block contains three convolutional layers; therefore, there are 49 convolutional layers in total, that is, 1 + 3 × (3 + 4 + 6 + 3) = 49 (**Figure 1**). The structure is as follows:

The size of the input data of the ResNet50 neural network is 224 × 224 × 3. After the image passes through the continuous convolution operation of the residual blocks, the channels of the pixel matrix of the image become deeper and deeper. Subsequently, after passing through the Flatten layer, the size of the image pixel matrix is changed to 2048. Finally, it is input into the fully connected layer, and the corresponding category probability is output through the SoftMax layer. The ResNet50 structure contains cross-layer connections, which pass the input across layers through shortcuts, and then adds the output after convolution to fully train the underlying network. As a result, the accuracy is significantly improved with the increase in depth (**Figure 2**). The structure of the residual block of the ResNet is as follows:

The shortcut connection, as seen in the figure above, has a function equivalent to performing equivalent mapping directly. However, this operation does not add any additional parameters, nor does it lead to computational complexity. Therefore, the model is reduced to a shallow network to a certain extent. To avoid this, the identical mapping function H(x) = x must be learned, but directly fitting such a function is challenging. Let us suppose that the output of the residual network is H(x) and the output after the convolution operation is F(x), H(x) = F(x) + x. For F(x) = (ω3δ(ω2δ(ω1x))), where ω refers to the convolution operation and δ represents the activation function. Therefore, if F(x) = 0, the aforementioned identical mapping function H(x) = x can be easily obtained, and the problem that needs to be addressed is learning an easily fitted residual function F(x) = H(x) - x.

### Model Development

Pytorch framework was used for model development. In the implementation of our model, an open-source repository is used (available at https://pytorch.org/). During training, we use ADAM as the optimizer, which is initialized with the learning rate of 1e-4. In the analyses of the results, we use the CAM technique implemented in an open-source repository (https://github.com/yizt/Grad-CAM.pytorch). The data were divided into a training set and a test set in a ratio of 8:2. ResNet50 was used as the classifier, and the original images were directly inputted into the network to achieve end-to-end prediction. While training the model, we divided the data set into five parts (No.1–No.5) and trained five models at the same time. No. 1 was considered as the test set for the first model, and the others were considered as the training set; No. 2 was considered as the test set for the second model, and the others were considered as the training set, and the rest were done in the same manner. In the evaluation phase, we recorded the performance of the model on the classification task of high and low expression of SYP. Taking high expression of SYP as a positive event, we obtained TP, TN, FP, and FN based on the confusion matrix and the following indicators were calculated. Sensitivity = TP/(TP + FN), Specificity = TN/(FP + TN), Positive predictive value = TP/(TP + FP), Negative predictive value = TN/(TN + FN), Accuracy = (TN + TP)/(TN + FP + FN +TP).

### Statistical Analyses

JMP 10.0 software (SAS Institute Inc., Cary, NC, USA) was used to conduct the statistical analyses. Comparison between groups was conducted using the chi-square test, Fisher’s exact test, or binomial distribution test for categorical variables, while the independent t-test for continuous variables. The Kaplan–Meier estimate and Cox proportional-hazards regression model were used for the survival analysis. The P value less than 0.05 is deemed as statistically significant.

## Results

### Clinical Significance of SYP

**Figure 3:**
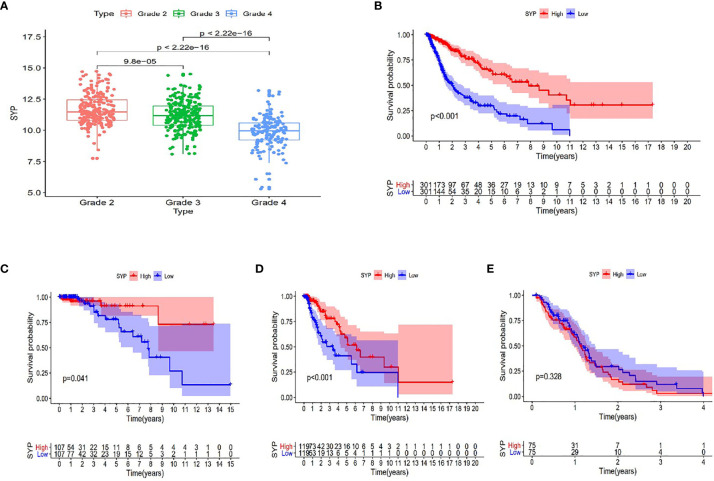
Expression of SYP genes in different grades of gliomas and their relationship with the survival rate of patients. **(A)** Expression level of SYP genes is significantly correlated with the grade of gliomas. **(B–E)** In terms of patients with grade II, III and the overall, the higher the level of SYP expression, the higher the survival rate of patients, while in terms of patients with grade IV, the level of SYP expression is not related to prognosis.

After analyzing the sequencing data of 614 cases from TCGA, we found the expression level of SYP in the glioma to be correlated with the tumor grade and the survival rate of the patient, and that the expression level of SYP decreased with an increase in tumor grade (**Figure 3A**). In glioma patients, particularly in grade II and grade III patients, the higher the expression level of SYP, the better was the survival rate of the patient (**Figures 3B–D**). In grade IV patients, the expression level of SYP was not associated with the survival rate (**Figure 3E**). It is suggested that SYP can be a molecular index to judge the tumor grade and predict prognosis, especially for low-grade gliomas.

### Molecular Markers and SYP

**Figure 4:**
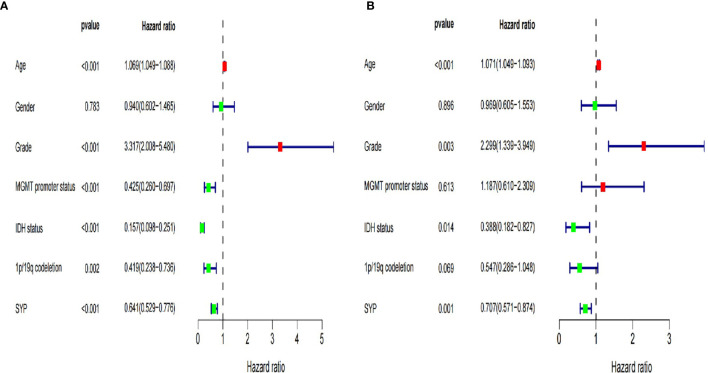
Forest map of clinical characters in univariate **(A)** and multivariate analysis **(B)**. The coordinate of the blue diamond represents the odds ratio. Univariate and multivariate Cox regression analysis were performed. Subgroup with a value of *p* < 0.05 was considered statistically significant.

At the same time, we verified the expression levels and status of well-known molecular markers including MGMT promoter methylation, IDH1 mutant, and co-deletion of 1p19q in low-grade glioma patients (WHOII, WHOIII) between high and low expression of SYP (**Supplementary Figures S1A–C**). A total of 288 patients had co-deletion of 1p19q, and the expression level of SYP was 11.7 ± 0.068; 169 patients had no co-deletion of 1p19q and the expression level of SYP was 10.93 ± 0.0886, p < 0.0001. It prompted that the expression level of SYP in the patients with common deletion of 1p19q was higher (**Supplementary Figure S1C**). Among the patients of the WHOII level, 160 patients had MGMT promoter methylation, and the expression level of SYP was 11.82 ± 0.0915; 31 patients had no MGMT promoter methylation, and the expression level of SYP was 11.48 ± 0.2512, p = 0.15. There was no significant difference between the two. It was shown that the expression level of SYP was higher in patients with MGMT promoter methylation. Among the patients of the WHOIII level, 195 patients had MGMT promoter methylation, and the expression level of SYP was 11.32 ± 0.0659; 49 patients had no MGMT promoter methylation, and the expression level of SYP was 10.68 ± 0.2058, p = 0.006. Among patients with WHOIII gliomas, it was shown that the expression level of SYP was higher in patients with MGMT promoter methylation (**Supplementary Figure S1A**). Among the patients of the WHOII level, the IDH genes of 19 patients were of the wild type and the expression level of SYP was 11.91 ± 0.399; the IDH genes of 198 patients were mutant and the expression level of SYP was 11.67 ± 0.082, p = 0.46. There was no significant difference between the two. Among the patients of the WHOIII level, the IDH genes of 67 patients were of the wild type and the expression level of SYP was 10.47 ± 0.169; the IDH genes of 177 patients were mutant and the expression level of SYP was 11.46 ± 0.715, P< 0.0001. It was shown that the expression level of SYP was higher in patients with mutant IDH genes (**Supplementary Figure S1B**). In order to make clear the influence of related genes on prognosis, we performed a regression analysis of a single factor and multi-factors (**Figure 4**) (**Supplementary Figures S1D, E**).

### Analysis of Predictive Results of a Neural Network Model

Based on the good predictive performance of the SYP gene in low-grade gliomas, preoperative MRI data of 124 patients with WHO grades II and III were downloaded from the TCGA database. Among them, 4 patients who lacked sequencing results and 12 patients who lacked complete T1, T2, and T1 enhanced phase sequences were excluded. A total of 108 patients were selected and grouped according to their the previous median SYP expression. There were 48 cases with high SYP expression and 60 cases with low SYP expression. There was no significant difference in sex and age between the group with high expression of SYP and the group with low expression of SYP.

**Figure 5:**
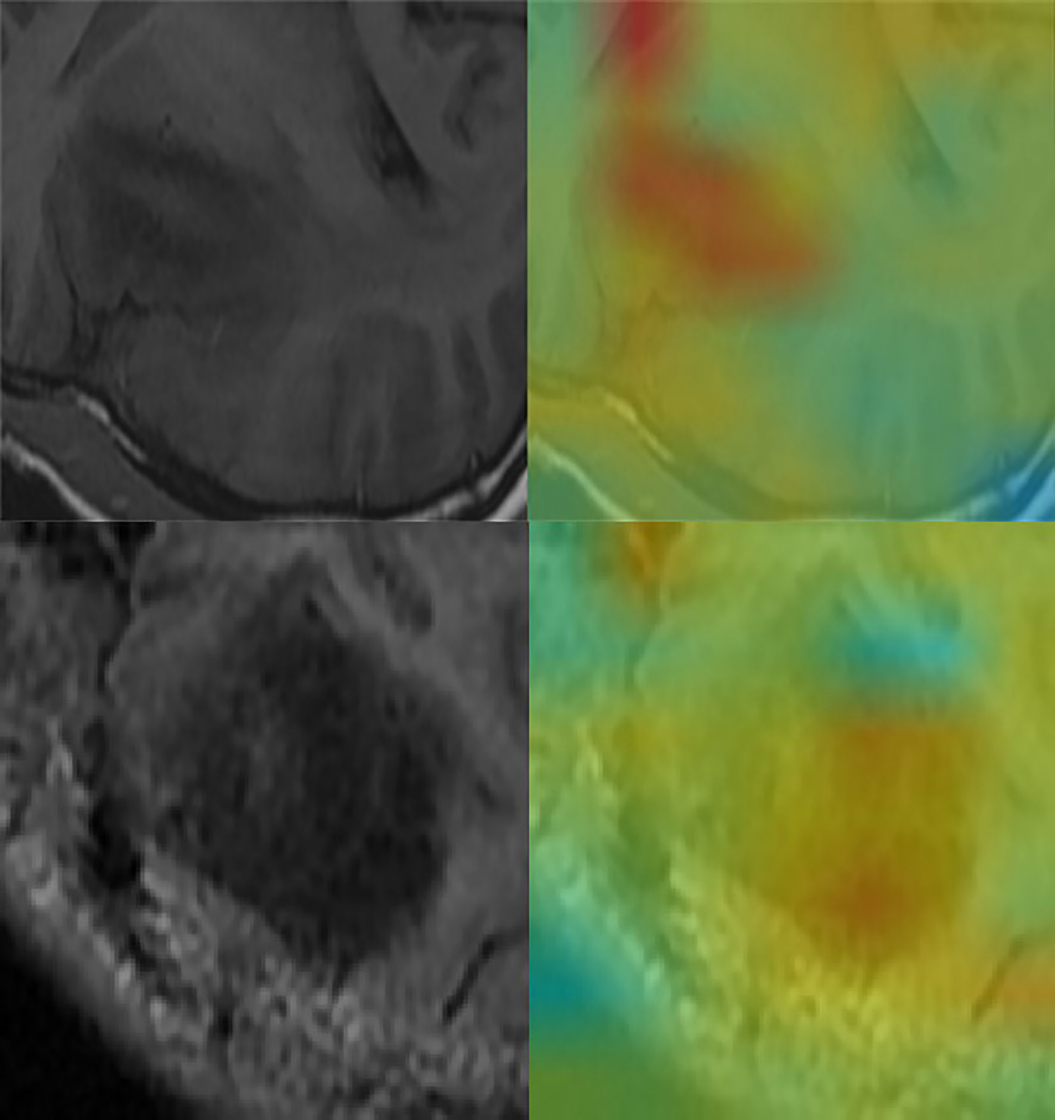
Convolutional neural network for the extraction of image features. Through the automatic extraction of image features by class activation mapping (CAM), the areas marked red in the image are the ones with high activation response to the visualized image.

**Figure 6:**
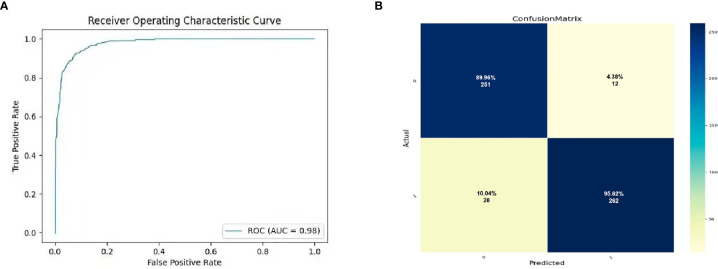
The prediction potential of convolutional neural network for the expression level of SYP genes. **(A)** Evaluation of radiomics model constructed by convolutional neural network through ROC. **(B)** Confusion matrix of the radiomics model. The upper left is true negative, the lower left is false negative, the upper right is false positive, and the lower right is true positive.

MRI images were classified according to median SPY values. Those greater than the median were considered positive, and those lower than the median as negative. After picking out the images with tumor regions and classifying them by cross-section, 3822 positive patches and 3444 negative patches were obtained (**Figure 5**). The model trained 250 rounds in total. The ROC curve, accuracy, positive predictive value, negative predictive value, sensitivity, and specificity were used as evaluation indexes. For the prediction model in the test group, the ROC curve area = 0.98 (**Figure 6A**), accuracy = 0.93, sensitivity = 90.34%, specificity = 95.44%, positive predictive value = 95.62%, and negative predictive value = 89.96% (**Figure 6B**).

## Discussion

Glioma, a type of malignant tumor originating from neuroglial cells, is one of the most common primary intracranial tumors (17). Grade II and III gliomas are regarded as low-grade gliomas that are well-differentiated, slow-growing, and biologically less invasive (18). However, they usually show significantly different clinical manifestations, recurrence rates, and prognosis (19). According to previous studies, patient age (>40 years), tumor resection, and tumor histology classification are important predictors of poor prognosis in low-grade gliomas (20–23). Nevertheless, Daniel J Brat used the TCGA database to divide LGGs into three categories based on isocitrate dehydrogenase mutation and 1p/19q gene deletion state in 2015 (2), including neuroglioma with IDH mutation and 1p/19q gene deletion, neuroglioma with IDH mutation, and without 1p/19q gene deletion, and neuroglioma with wild-type IDH. Furthermore, it was found that the new classification scheme could be more precise in reflecting the biological characteristics of LGGs, instructing patient treatment, and predicting prognostic status than the traditional classification (24); therefore, the significance of molecular biomarkers has attracted widespread attention (1).

In accordance with clinical work, synaptophysin (SYP) can be used as a predictor of disease progression and clinical prognosis of gliomas, especially low-grade gliomas (7). Unlike the malignant progression of glioblastoma, there is a great heterogeneity in the prognosis of patients with low-grade gliomas, ranging from one or two years to more than ten years. Therefore, it is highly significant to make a personalized and accurate prediction of the prognosis of patients with low-grade gliomas. The expression results of SYP, which is a common index for the pathological diagnosis of glioma, are easy to obtain. Further, it is simple, rapid, and highly effective for evaluation of prognosis of patients.

However, traditional CT and MR imaging techniques cannot be applied to the molecular diagnosis of gliomas, and the rise of imaging technology makes the connection between machine learning and molecular diagnosis possible (25). This study adds ConvNet technology to the traditional machine learning method. Consequently, the considerable improvement in image processing enables automated feature extraction, filters characteristics free from manual design, and avoids subjective results, eventually acquiring a better predictive performance. This is the core advantage of model building suggested in this study.

There are some limitations to this study. First, the input images are only tomographic MRI, which might enhance the predictive performance of the study’s model further in case of segmentation in the coronal plane, sagittal plane, or other multilevel reconstruction of images. Second, the study includes relatively few cases, so the inclusion of more data to further enhance the accuracy and universality of the ConvNet model is suggested.

In conclusion, the ConvNet model built in this study is able to discern the expression level of glioma SYP impartially and effectively. In consideration of a better predictive result, the ConvNet model is groundbreaking in the development of a multi-parameter model to help enhance the individualized diagnosis and treatment of gliomas.

## Data Availability Statement

Publicly available datasets were analyzed in this study. This data can be found here: TCIA(https://www.cancerimagingarchive.net/) TCGA(http://cancergenome.nih.gov) The model is stored in the Baidu network disk: https://pan.baidu.com/s/1AmXjAQb5Oyt_9LGQiUNbTw Password.99yc.

## Ethics Statement

Ethical approval was not provided for this study on human participants because the imaging data and TCGA sequencing data were downloaded from the TCIA and the TCGA. As the patients’ private information were de-identified by the TCGA/TCIA organization and their information was made available for download by public, we did not have to apply for the approval of the Institutional Review Board or the health organizations following the Health Insurance Portability and Accountability Act. Written informed consent from the participants’ legal guardian/next of kin was not required to participate in this study in accordance with the national legislation and the institutional requirements.

## Author Contributions

Conceived and designed the experiments: ZX and W-lC. Analyzed the data: Z-mW, D-mZ, and Y-nB. Wrote the paper: ZX and Y-nB. Edited and revised the manuscript: Y-nB, S-zZ, and W-lC. Improve the model: SY. All authors contributed to the article and approved the submitted version.

## Funding

This work was supported by Guangdong Medical Research Foundation (A2018245), Natural Science Foundation of Guangdong province (2018A0303130333), Medical Scientific Research Foundation of Guangdong Province, China (A2020449), Chinese Postdoctoral Science Foundation (2019M663271) and Guangdong Enterprise Science and Technology Commissioner Project (GDKTP2020041900).

## Conflict of Interest

The authors declare that the research was conducted in the absence of any commercial or financial relationships that could be construed as a potential conflict of interest.

## Abbreviations

SYP, Synaptophysin; LGG, low grade glioma; MRI, Magnetic resonance imaging; TCGA, The Cancer Genome Atlas; TCIA, The Cancer Imaging Archive; ROC, Receiver Operating Characteristic; PRC, precision recall curve; ROI, region of interest; GBM, Glioblastoma multiform; ConvNet/CNN, convolutional neural network; MGMT,O-6-methylguanine-DNA methyltransferase; IDH, Isocitrate dehydrogenase; TR, Repetition time; TE, Echo time; FOV, Field of view.
