# Supplementary material for: Multiparametric MRI Features Predict the SYP Gene Expression in Low-Grade Glioma Patients: A Machine Learning-Based Radiomics Analysis
Source: Front Oncol. 2021 May 31;11:663451. doi: 10.3389/fonc.2021.663451 (PMC8202412; doi:10.3389/fonc.2021.663451)
Supplement: Supplementary file 1 [file Image_1.pdf]

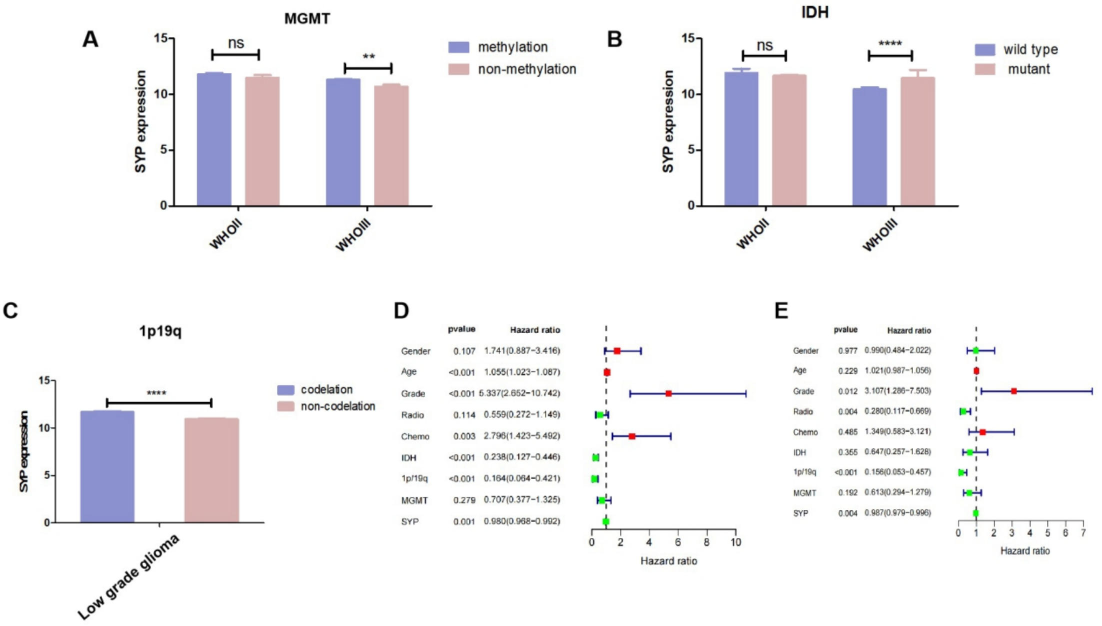

Supplementary Figure S1. Forest map of clinical characters in univariate and multivariate analysis and correlation analysis of MGMT, IDH1, 1p19q and SYP. (A--C) The expression levels and status of MGMT, IDH1 and 1p19q in low-grade glioma patients (WHOII, WHOIII) between high and low expression of SYP. (D--E) Influence of molecular markers and clinical features on prognosis of patients with low grade glioma, (D) Univariate analysis, (E)Multivariate analysis. p < 0．05，**p < 0．01，***p < 0．001，****p < 0．0001，ns: no significance.
